# Energetic Effects of Pre-hatch Albumen Removal on Embryonic Development and Early Ontogeny in *Gallus gallus*

**DOI:** 10.3389/fphys.2016.00690

**Published:** 2017-01-10

**Authors:** Isaac Peña-Villalobos, Gabriela Piriz, Verónica Palma, Pablo Sabat

**Affiliations:** ^1^Laboratorio de Ecofisiología Animal, Departamento de Ecología, Universidad de ChileSantiago, Chile; ^2^Laboratorio de Células troncales y Biología del Desarrollo, Departamento de Biología, Universidad de ChileSantiago, Chile; ^3^Facultad de Ciencias Biológicas, Center of Applied Ecology and Sustainability, Pontificia Universidad Católica de ChileSantiago, Chile

**Keywords:** citrate synthase, cytochrome c oxidase, hatching, maternal effect, metabolic rate

## Abstract

Studies on the yolk and albumen content in bird eggs, and the effects of variations in their relative loads in the phenotype of the birds, have revealed multiple consequences at different levels of biological organization, from biochemical traits to behavior. However, little is known about the effect of albumen variation on energetics performance during development and early ontogeny, despite the fact that variation in energy expenditure may have consequences in terms of fitness for both feral and domestic species. In this work, we evaluated experimentally whether variations in the content of albumen of *Gallus gallus* eggs could generate differences in metabolic rates during embryonic development. Additionally, we assessed changes in the activity of mitochondrial enzymes (cytochrome c oxidase and citrate synthase) in skeletal muscles and liver. Finally, we evaluated the success of hatching of these embryos and their metabolic rates (MR) post-hatching. The results revealed a significant reduction in MR in the last fifth of embryonic life, and reduced catabolic activities in the skeletal muscle of chicks hatched from albumen-removed eggs. However, the same group demonstrated an increase in catabolic activity in the liver, suggesting the existence of changes in energy allocation between tissues. Besides, we found a decrease in hatching success in the albumen-removed group, suggesting a negative effect of the lower albumen content on eggs, possibly due to lower catabolic activities in skeletal muscle. We also found a compensatory phenomenon in the first week after hatching, i.e., birds from albumen-removed eggs did not show a decrease in MR either at thermoneutral temperatures or at 10°C, compared to the control group. Collectively, our data suggest that a reduction in albumen may generate a trade-off between tissue metabolic activities, and may explain the differences in metabolic rates and hatching success, supporting the immediate adaptive response (IAR) hypothesis.

## Introduction

During embryonic development, animals may demonstrate a more than 100-fold increase in body mass, with a paralleled dramatic rise in metabolic rate (Vleck and Vleck, 1980). This phenomenon requires both the availability of biomolecules for maintaining high rates of biosynthesis, as well as high rates of energy expenditure to maintain these new structures (Rombough, 2006; Mortola and Cooney, 2008). However, embryonic development is a dynamic process affected not only by the genetic background, but also by the environment in which it develops (De Smit et al., 2006). It is well-known that a reduction in the supply of nutrients may have deleterious consequences for embryonic development and biological performance in more advanced stages of ontogeny. These ideas derive from observations in human fetal malnutrition that were observed following the “Dutch Hunger Winter” of 1944–1945 in the Second World War (Stein, 1975; Hart, 1993), a period in which several hypotheses were formulated to explain the effects of pre-natal undernutrition (Gilbert and Epel, 2009). For instance, it has been hypothesized that nutrient-poor embryonic environments generate compromises in fetal development through energy reallocations (trade-offs) between organs or tissues (e.g., brain growth at the expense of non-vital organs). This hypothesis has been referred to as the immediate adaptive response (IAR; Bateson et al., 2004). Furthermore, a second hypothesis, called predictive adaptive response (PAR), postulates that environmental cues encountered during early life may modify embryonic development in order to cope with predicted environmental conditions in later life.

With regards to the nutritional sources required for embryonic development, in albumen and yolk, avian eggs contain all components necessary for this process. Yolk contains a high and variable proportion of fat, hormones, vitamins and antibodies, and serves as the primary source of energy, while proteins and water found in albumen are used to form the structures of the embryo (Romanoff and Romanoff, 1949; Royle et al., 1999; Groothuis and Schwabl, 2002; Staszewski and Siitari, 2010; Ho et al., 2011; Parolini et al., 2015). Interestingly, some studies have reported natural variation in the proportion of albumen content in eggs of wild birds (Christians and Williams, 2001; Christians, 2002; Dzialowski and Sotherland, 2004; Ferrari et al., 2006; Alquati et al., 2007). Thus, egg size variation in relation to laying order has been extensively investigated as a pattern of a positive relationship between egg size and offspring performance, due to the fact that large eggs contain more reserves and result in larger chick size at hatching (Ferrari et al., 2006). It has been reported that egg mass varies according to laying order in a clutch, but the relationship differs not only between species (i.e., increasing or diminishing egg mass) but also within conspecific bird populations and over time (Ojanen et al., 1981; Slagsvold et al., 1984; Wiebe and Bortolotti, 1996; Ferrari et al., 2006). Within-species variation in egg mass is known to depend mainly on variation in albumen rather than yolk mass (Sotherland et al., 1990; Hill, 1995). This variation in the proportion of albumen is an example of a maternal effect, and is considered a purposeful force that helps shape the phenotype of offspring (Mousseau and Fox, 1998; Bateson et al., 2004; van der Waaij et al., 2011; Willems et al., 2016).

This natural variation in albumen content in eggs can be simulated through albumen removal, allowing the potential effects, including the consequences for individual fitness, to be evaluated at a physiological level. Commonly, experimental manipulations for the purposes of studying malnutrition involve removal of either albumen or yolk via a hole in the egg shell (Finkler et al., 1998; Ferrari et al., 2006; Alquati et al., 2007; Bonisoli-Alquati et al., 2008; Everaert et al., 2013; Willems et al., 2013, 2014a,b, 2015). Several studies have identified changes generated by the removal of albumen in early ontogeny (after hatching), including time to hatching, begging behavior, reductions in body mass, decreased plasma T4, high plasma uric acid concentration, upregulation of TCA cycle enzymes in liver, morphology, survival, and hatching asynchrony (Finkler et al., 1998; Ferrari et al., 2006; Alquati et al., 2007; Bonisoli-Alquati et al., 2008; Willems et al., 2014b). Regarding responses generated in later ontogeny (juvenile growth and adulthood), an increase in immune response, higher mRNA expression of muscle ring finger-1 (*MuRF1*, a gene related to proteolysis), reduction of body mass, decreased egg production accompanied by higher proportion of yolk/ albumen, and long-term alteration of the hepatic transcriptome in chickens have been reported (Everaert et al., 2013; Willems et al., 2013, 2015, 2016).

To date, only two studies have explored energetic effects of protein deprivation, and their results provided somewhat conflicting evidence for the existence of metabolic changes. For instance, Finkler et al. (1998) found no effects of protein deprivation on total metabolic rate in chicken embryos at day 18. However, Willems et al. (2014b) reported a reduction in plasma thyroxin (T4) at day 20 of embryonic development (E20), suggesting a reduction in metabolic rate, along with an increase in abundance of enzymes associated with catabolism in the liver.

The importance of analyzing metabolic rate stems from the fact that it is a fundamental measure in physiological ecology (Brown et al., 2004) and linked to the survival of both feral and domestic species. The study of energetic performance and reallocation of matter and energy within an organism under nutritional shortage therefore represents an opportunity to advance current scientific understanding of animal physiological responses to external factors (e.g., maternal effects) and their consequences for fitness (e.g., survival or reproductive success).

The main objective of this study was to analyze experimentally the metabolic effects of albumen removal in a precocial bird. Accordingly, we simulated differential egg size composition observed in relation to laying order in an attempt to understand the energetic consequences of protein deprivation during the stages of embryonic development, hatching, and early ontogeny post-hatching. We hypothesized that a decrease in the embryonic protein source, by means of the removal of a portion of albumen, could generate differences in metabolic rates during embryonic development, due to reallocations of energy and nutrients between tissues (i.e., supporting the IAR hypothesis). Further, we expected these changes to produce effects in early ontogeny due to trade-offs generated during embryonic growth.

To evaluate this hypothesis, we analyzed the aerobic metabolism from E4 to hatching, and metabolic enzymes (citrate synthase and cytochrome c oxidase) in pectoral muscles, lower limb, and liver, in order to identify at which embryonic age differences in metabolic cost are established, and to assess whether trade-offs in the development of the metabolic activity of tissues occur. We also evaluated differences in hatching rates, in order to analyze whether the variation in the amount of albumen could reduce muscular ability to break the shell at hatching. Finally, we aimed to determine both resting metabolic rates and metabolic rates under a thermoregulatory challenge in 1 week old chicks, to analyze the potential energetic consequences on early ontogeny.

## Materials and methods

We choose *Gallus gallus* as a model of study because this species is of common use in studies of embryonic development in birds. Specifically we used the broiler strain (Ross) because it exhibits an advanced thermogenic capacity, a more efficient use of nutrients sources and higher oxygen consumption during development than layer strains (Buzała et al., 2015).

### Removal albumen and incubation

Three hundred and ninety fertilized broiler eggs were purchased from a local supplier and were randomly separated into experimental, control and sham groups. Eggs of the three groups displayed no significant differences in terms of mass [control: 47.27 ± 7.66 g, experimental: 49.36 ± 5.10 g, and sham: 50.30 ± 4.10 ANOVA: *F*_(2, 24)_ = 2.95, *p* = 0.071] length [control: 57.02 ± 1.10 mm, experimental: 58.95 ± 3.50 mm, and sham: 58.23 ± 4.10 mm ANOVA: *F*_(2, 24)_ = 2.59, *p* = 0.083] or diameter [control: 42.18 ± 1.55 mm, experimental: 44.98 ± 3.15 mm, and sham: 41.53 ± 2.10 mm ANOVA: *F*_(2, 24)_ = 2.97, *p* = 0.081]. Given that it demonstrates a positive linear relationship with egg size (Finkler et al., 1998), we assume no difference between the eggs in terms of albumen content. All eggs were cleaned using a solution of 70% ethanol applied with a towel. At 2 days of embryonic development (E2), holes were carefully drilled on blunt end of eggs from the experimental group, and 3 ml of albumen extracted by means of sterile 5 mL disposable syringes (Willems et al., 2014b, 2015). This volume corresponds nearly to 10% of the total volume (average volume 32.49 cm^3^). We estimated that 3 mL of albumen contains ~0.31 g protein, according to Meuer and Egbers (1990) and Steven (1991). Immediately, the same amount of Ringer sterilized solution was introduced to prevent dehydration. Holes were sealed with a piece of adhesive tape of between 3 and 5 mm^2^. The sham group was mock-treated, similar to the albumen-removal group, except for the actual albumen removal and Ringer solution injection. All eggs were incubated at 38°C ± 0.5 in a circulated air incubator (G.Q.F. MFG. Co., USA), with a relative humidity of 50 to 60% until E18. Then we increased the humidity of incubator until 90% RH to facilitate hatching.

### Resting metabolic rate

Whole egg metabolic rates (embryo + extraembryonic membranes) in different cohorts (*n* = 6) of eggs during (E4), (E8), (E12), (E16), and (E20) were estimated as the oxygen consumption (V_O2_) using standard flow-through respirometry methods. Because VO_2_ at E4 was too low to be detected by the oxygen sensor, at this stage the CO_2_ production (V_CO2_) was measured to estimate metabolic rate. Then, in E4 the O_2_ consumptionwas calculated using the respiratory quotient (V_CO2_/V_O2_) of 0.71 (Walsberg and Wolf, 1995). From E8 to E20 the V_O2_ was measured directly. Eggs were briefly weighed and placed in a transparent acrylic chamber (300 mL) located in a temperature controlled dark cabinet (Sable Systems, Henderson, Nevada), with a constant ambient temperature (Ta = 37.5 ± 0.5°C). The metabolic chamber received air dried at 130 mL min^−1^ from a mass flow controller and-through Bev-A-Line tubing (thermophilus moplastic Processes Inc.). The excurrent air passed-through columns of Drierite, CO_2_-absorbent granules of Baralyme and Drierite before passing-through an O_2_ analyzer, model Turbo Fox (Sable Systems, Henderson, Nevada) calibrated with a known mix of oxygen (20%) and nitrogen (80%), concentrations of which were certified by chromatography (BOC, Chile). The mass flow meter of the Turbo Fox was calibrated monthly with a volumetric (bubble) flow meter. The measurement and calibration protocols followed those of Williams and Tieleman (2000). Because water vapor and CO_2_ were scrubbed before entering the O_2_ analyzer, oxygen consumption was calculated as Withers (1977, p 122): VO_2_ = [FR × 60 × (Fi O_2_ − O_2_ Fe)]/(1 − Fi O_2_), where FR is the flow rate in ml/min STP after correction, and the Fi and Fe are the fractional concentrations of O_2_ entering and leaving the metabolic chamber, respectively. The CO_2_ signal (for E4) was transformed to flow units (mL h^−1^) with the relationships: CO_2_ = 60·FR·CO_2_/100 (Withers, 1977). Output from the oxygen and carbon dioxide analyzer (%) and flow meter were digitalized using a Universal Interface II (Sable Systems) and recorded on a personal computer using EXPEDATA data acquisition software (Sable Systems). Our sampling interval was 1 s, with measurements performed over periods of 30 min or until a plateau in oxygen consumption levels was obtained.

### Organ mass

After metabolic determinations, animals of all embryonic stages were removed from the eggs and sacrificed by decapitation over a cold surface. Organs (liver, eyes, heart, and gizzard), chorioallantoic membrane (CAM), and yolk sac and contents (YS) were removed and weighed (±0.0001 g); they were subsequently dried to attain constant mass (typically after 7 days). These organs were analyzed because some (e. g., eyes and heart) exhibit negative allometry in growth, and therefore have a high contribution of biomolecules in their early development, while others demonstrate high-energy consumption (e. g., liver and CAM).

### Enzyme determinations

After metabolic determinations, E20 embryos were sacrificed as described. Organs (liver, intestine, heart, and gizzard), CAM and yolk sac were removed and weighed (±0.0001 g). Animals were dissected on ice and the liver, pectoralis, and supracoracoideus were extracted, along with the major muscles of both lower limbs (iliotibialis cranialis, iliotibialis lateralis, iliofibularis, femorotibialis externus, fibularis longus, tibialis cranialis, extensor digitorum longus, and gastrocnemius complex) (Liknes and Swanson, 2011a; Peña-Villalobos et al., 2014). For the analyses we used the homogenates of the entire flight muscles (pectoralis plus supracoracoideus = pectoral muscles), the set of muscles of the lower extremities (leg muscles) and the liver. The tissues were stored at −80°C for further enzyme assays.

Tissues were thawed, weighed, and homogenized in 10 volumes of phosphate buffer 0.1 M with EDTA 0.002 M (pH 7.3) with an Ultra Turrax homogenizer (20,000 rpm) on ice to avoid enzymatic reactions. Samples were then sonicated at 130 watts for 20 s at 10 s intervals, 14 times each, using an Ultrasonic Processor VCX 130, while maintained on ice. Cellular debris were removed by centrifugation for 15 min at 12,000 g and 4°C. The supernatant was carefully transferred into a new tube, avoiding co-transference of the upper lipid layer present in the liver preparations. Protein concentration of the samples was determined by the method described by Bradford (1976), using bovine serum albumin as standard. Activities of two mitochondrial enzymes were determined: cytochrome c oxidase (COX; E.C. 1.9.3.1), and citrate synthase (CS; E.C. 4.1.3.7). COX activities were determined spectrophotometrically according to Moyes et al. (1997), with slight modifications. Enzyme activity was determined in 10 mM Tris/HCl pH 7 containing 120 mM KCl, 250 mM sucrose, and cytochrome c reduced with dithiothreitol to a final volume of 0.2 ml. The decrease in D.O. at 550 nm was monitored in a thermo Scientific Multiskan GO spectrophotometer at 25°C. Enzyme activity in units per gram of wet tissue was calculated using an extinction coefficient of 21.84 mM^−1^cm^−1^ at 550 nm for COX. CS activities were measured according to Sidell et al. (1987) with small modifications. The CS assay medium contained 10 mM Tris/HCl, pH 8.0, 10 mM 5,5′dithiobis-(2·nitrobenzoic acid) (DTNB), 30 mM acetyl Coenzyme A (Acetyl CoA) and 10 mM oxaloacetic Acid (OAA; omitted for the control) in a final volume of 0.2 mL. Citrate synthase catalyzes the reaction between acetyl CoA and OAA to form citric acid. The increase in O.D. at 412 nm was measured in a Thermo Scientific Multiskan at 25°C. Enzyme activity was calculated using an extinction coefficient of 13.6 mM ^−1^cm^−1^ at 412 nm. Enzyme activities are reported as mass-specific activities (μmol min^−1^ g fresh mass^−1^), mean total activity (μmol min^−1^), and specific activity per gram of protein (μmol min^−1^ g protein^−1^).

### Hatching comparison

To compare hatching rates, sham, control, and experimental eggs (70 eggs per treatment) were incubated for 21 days under identical temperature and humidity conditions. No assistance was provided to hatchlings in breaking the shell. Only chicks that left the shell within at least 2 days after starting piping were considered hatched.

### Post-hatch analysis

Newly hatched birds were kept in the same room indoors, with food and water *ad libitum*, at a temperature of 30°C and photoperiod of L:D 12:12. Birds were weighed daily, and by day 7 post-hatch, resting metabolic rate was determined by the same method used for eggs, except that the volume of the chamber was changed to 1 L and the air flow to 500 ml min^−1^. Animals were measured at an environmental temperature of 30°C ± 0.5 by 2 h. Furthermore, we determined the metabolic rate in a cold challenge, thus the oxygen consumption was analyzed at 0.5 h under the same conditions, at a temperature of 10°C ± 1.0 (Sable Systems, Henderson, Nevada). Following metabolic determinations, animals were sacrificed by decapitation and organs weighed and dried for determination of dry mass at a later stage.

### Statistical analysis

We calculated mass-specific resting metabolic rate considering both the wet and dry mass of embryos, since their mass is comprised of a high percentage of body water (preliminary analysis shows values between 83 and 96% water). Further, because CAM metabolism comprises a large fraction of total egg oxygen consumption during the E5–E12 period (Needham, 1932; Romanoff, 1967; Pearson et al., 1991), we also included the CAM mass in our calculations of the mass-specific resting metabolic rate. Thus, we express the mass-specific resting metabolic rate as total metabolic rate / (body mass + CAM mass). We compared metabolic rates by correcting by both body mass and metabolic body mass, i.e., considering the allometric exponent for pre-hatching (mb^0.86^) birds (Klaassen and Drent, 1991). Morphological, metabolic, and biochemical data for the different groups were compared using a permutation test (Pt) of differences based on 10,000 permutations, performed in R (R Development Core Team, 2009). For each treatment, we evaluated potential associations between physiological, morphological, and biochemical variables by means of Pearson correlations, performing permutation tests (Pt) for the coefficients of correlation (*n* = 10,000). In cases where morphological and physiological variables were correlated with either body or egg mass, their residuals were used to perform the correlation analysis. The hatching comparison was performed using a chi-square test to evaluate the relationship between treatment type and number of births, corrected by no fertilized or developed eggs. Statistical analyses were performed using the STATISTICA (2004) statistical package for Windows, and “R” version 3.1.2. for Windows.

This study was carried out in accordance with the recommendations of the guide “Regulation of the use and care of experimental animals” of the Bioethics Committee, Comisión Nacional de Investigación Científica y Tecnológica (CONICYT). The protocol was approved by the Institutional Animal Care Committee of the University of Chile.

## Results

### Embryonic organs mass and energetics

Experimental treatment had no effect on embryo and organ mass throughout embryonic development when variables were corrected by total egg mass, with the exception of leg mass, which was higher in the control group (see Table 1).

**Table 1:** **Comparison of body and organ masses corrected by egg mass, in *Gallus gallus* embryos of different ages, from eggs with albumen removed, control and sham group (*n* = 6 per group)**.

**Variables**	**Group**	**E4**	**E8**	**E12**	**E16**	**E20**
Body mass (wet)	Control	0.98 ± 1.0	15 ± 1.0	62 ± 12	199 ± 22	539.4 ± 66
	Albumen-removed	0.51 ± 13	19 ± 8.0	98 ± 26	270 ± 65	577 ± 59
	Sham	0.45 ± 0.1	15 ± 2.0	71 ± 8.0	222 ± 15	591.8 ± 88
Body mass (dry)	Control	0.041 ± 0.1	1.0 ± 0.1	5.0 ± 1.0	23 ± 3.0	99 ± 19
	Albumen-removed	0.056 ± 0.3	1.0 ± 1.0	9.0 ± 2.0	37 ± 12	110 ± 26
	Sham	0.045 ± 0.3	1.0 ± 1.0	6.0 ± 1.0	35 ± 3.0	94 ± 13
Eye	Control		1.3 ± 0.3	4.0 ± 0.3	6.4 ± 1.0	9.5 ± 1.0
	Albumen-removed		1.6 ± 1.0	5.0 ± 1.0	7.7 ± 1.0	8.7 ± 1.0
	Sham		1.6 ± 1.0	3.0 ± 0.3	7.6 ± 0.4	8.5 ± 1.0
Heart	Control			1.0 ± 0.1	2.4 ± 0.2	4.0 ± 0.1
	Albumen-removed			1.0 ± 0.3	2.7 ± 0.4	4.0 ± 1.0
	Sham			1.0 ± 0.3	2.2 ± 0.3	4.0 ± 1.0
Liver	Control			1.0 ± 0.4	4.8 ± 0.2	11 ± 1.4
	Albumen-removed			2.0 ± 0.6	6.1 ± 1.5	13 ± 4.0
	Sham			2.0 ± 0.1	6.9 ± 1.0	13 ± 2.0
Gizzard	Control			12 ± 0.5	9.1 ± 0.6	28 ± 9.0
	Albumen-removed			19 ± 8.1	9.8 ± 4.7	26 ± 6.0
	Sham			12 ± 2.0	9.9 ± 1.6	25 ± 9.0
Legs	Control					65 ± 8.0
	Albumen-removed					55 ± 6.0^*^
	Sham					60 ± 17

Values are dimensionless and are expressed as follows: [organ or body mass (g) / total egg (g)]

* *1000. Asterisk denotes statistical differences*.

**Figure 1:**
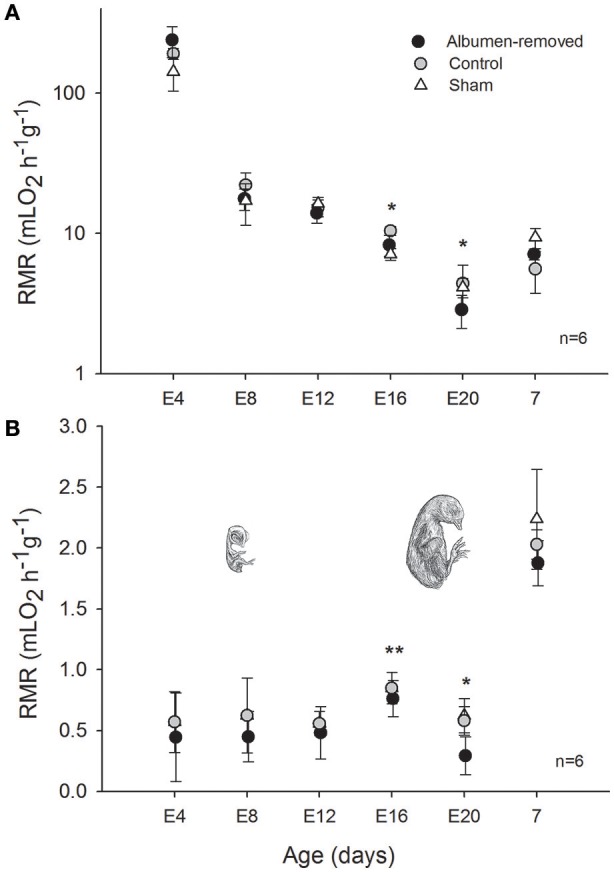
**Resting metabolic rate, corrected by dry, and wet body mass + CAM mass (A,B**, respectively), measured from E4 to 7 days after hatching in individuals of *Gallus gallus*, comparing eggs that underwent albumen removal (3 mL) at E2, vs. a control and sham group. We employ the respiratory quotient 0.71 to express the CO_2_ formed by O_2_ used. Asterisk indicates significant differences between groups (*p* < 0.05), ^**^indicate tendency (*p* = 0.053). Bars signify ± 1 *SD*.

**Figure 2:**
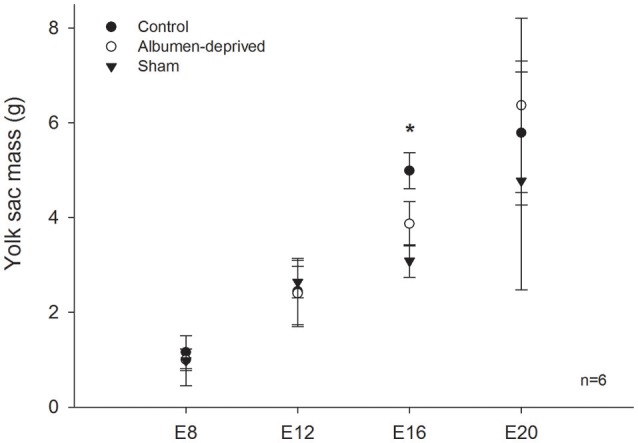
**Extra embryonic membrane mass in *Gallus gallus* embryos of different ages from eggs submitted to albumen removal vs. a control and sham group**. Comparison of yolk sac (with content). Asterisk (^*^) indicates significant differences between groups. Bars signify ± 1 *SD*.

Mass-specific resting metabolic rates of developing animals were statistically indistinguishable between all groups until E16, at which stage RMR was lower in the albumen-removed group relative to the control group and sham group (Pt; *p* = 0.002 and *p* = 0.031, respectively, Figure 1). Furthermore, we found that in E16 RMR/mb^0.86^ (based on dry masses) was 24% lower in albumen-removed individuals in comparison to the control and sham group (Pt; *p* < 0.001 and *p* < 0.001, respectively). When data were standardized by wet masses, RMR/mb^0.86^ was 9% lower, in the albumen-removed compared to the control and sham group (Pt; *p* = 0.040 and *p* = 0.035, respectively). In the E20 stage we found that RMR/mb^0.86^ (using dry masses) was 24% lower in albumen-removed individuals than the control and sham group (Pt; *p* = 0.0198 and *p* = 0.045, respectively). When we analyzed the data using wet masses we found that RMR/mb^0.86^ was 17% lower in albumen-removed individuals than the control and sham group (Pt; *p* = 0.0194 and *p* = 0.038, respectively). Finally, in E20 we found a significant and positive relationship between the residuals of RMR and the residuals of liver (*r*^2^ = 0.499; *r* = 0.704, *p* = 0.001). Regarding the extra-embryonic membranes, we did not find differences in mass of CAM or VM, except at E16, where the albumen-removed group had lower mass of YS (including yolk content; Figure 2).

### Enzymatic activities in E20

In line with the reported difference in leg mass, we found a strong effect of treatment on enzymatic activities of skeletal muscles. In pectoral muscles, total and mass-specific activity of COX was 1.5-fold lower in the albumen-deprived group than the control group (*p* = 0.0211 and *p* = 0.036, respectively), despite the sham group presented intermediate values (sham and albumen-removed *p* = 0.034; sham and control group *p* = 0.048). The enzymatic activity of CS was greater in leg muscles of the control and sham group, through the total activity (1.8-fold; *p* = 0.001 and *p* = 0.032, respectively) and mass specific activity (*p* = 0.012 and *p* = 0.022, respectively). Besides at mass-specific activity, albumen-removed embryos present a lower activity (*p* < 0.001). Additionally, the quotient of activity in pectoral muscles/activity in leg muscles was lower (0.3 ± 0.09) in the control group than in the experimental group (0.6 ± 0.24; *p* = 0.014).

Despite the observed decrease in enzyme activity in the skeletal muscles of albumen deprived group, we found that the livers of albumen-removed embryos displayed higher activity of CS and COX than control group, both on a mass-specific basis (CS: *p* = 0.027; COX: *p* = 0.020) and per gram of protein activity (CS: *p* = 0.023; COX: *p* = 0.022). The same pattern was found when we compared albumen-removed embryos with the sham group (CS: *p* = 0.025; COX: *p* = 0.031 and CS: *p* = 0.037; COX: *p* = 0.019, for mass-specific and per gram of protein activities, respectively).

**Figure 3:**
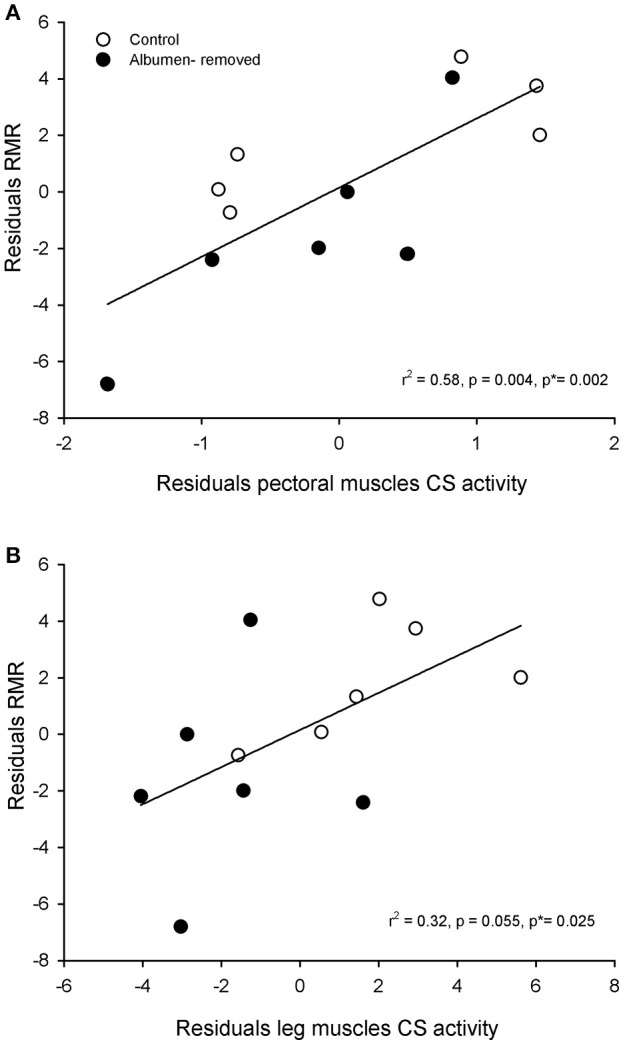
**Relationship between physiological parameters, in embryos (E20, *Gallus gallus*) from eggs that underwent albumen removal (3 mL) or control group**. **(A)**: correlation between residuals of citrate synthase activity of pectoral muscle and residuals of resting metabolic rate. **(B)**: correlation between residuals of citrate synthase activity of leg muscles and residuals of resting metabolic rate. P^*^ indicates the *p*-value obtained by permutation test (see text for details).

**Figure 4:**
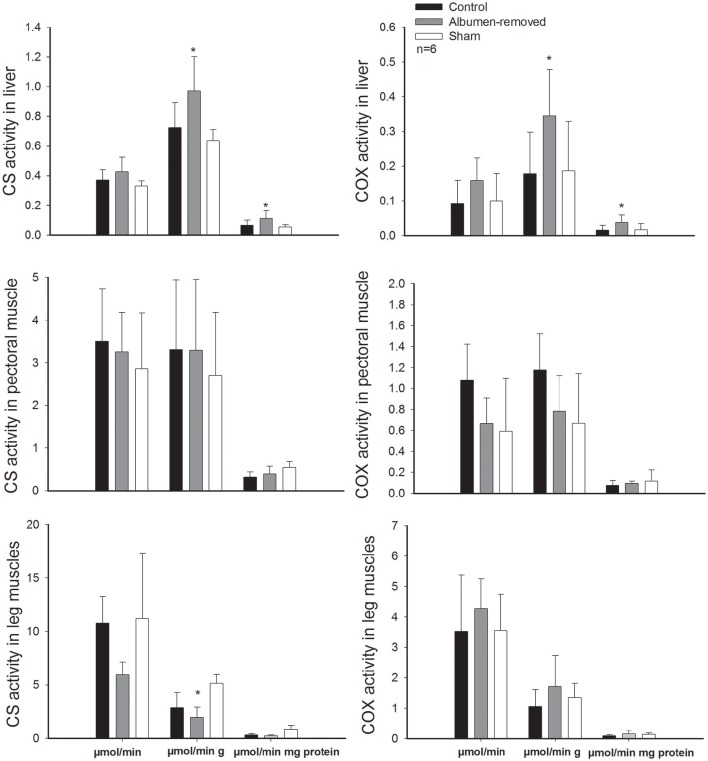
**Activity of the enzyme citrate synthase and cytochrome c oxidase in pectoral muscle, leg muscles, and liver, in embryos (E20, *Gallus gallus*) from eggs that underwent albumen removal (3 mL) vs. a control and sham group**. Asterisk denote differences in permutation tests (see text for details). Bars signify ± 1 *SD*.

Furthermore, we found a positive association between the residuals of RMR and residuals of CS in pectoral muscle (*r*^2^ = 0.576; *r* = 0.759, *p* = 0.004, permutation test *p* = 0.002, Figure 3A) and a trend in the association with CS in legs (*r*^2^ = 0.320; *r* = 0.565, *p* = 0.055), which showed significant association in the permutation test (*p* = 0.025, Figure 3B). All comparisons of enzymatic activities are presented in the Figure 4.

### Hatching comparison

Only 20% of embryos from the albumen removed group managed to break the shell and successfully hatch, with the sham and control groups showing greater hatching success (30 and 60%, respectively). The mortality of embryos of albumen removed group occurs with highly probability at the end of incubation (45% of embryos). Instead, in sham group a 62% of mortality occurs at beginning of embryo development. No temporal differences in mortality were observed in the control group. We found an association between the treatment and hatching success (χ^2^ = 6.690, df = 2, *p* = 0.034). Despite these differences at hatching, no effects of treatment on body mass of chicks were found (*p* < 0.05).

### Post-hatching chicks

We found no effects of treatment on the mass of chicks or in internal organs. Additionally, measures of metabolic rates at the two temperatures were similar among treatments at 7 days post-hatching (Table 2).

**Table 2:** **Comparison of metabolic rates and dry organs in *Gallus gallus* chicks (1 week old), contrasting albumen removed eggs with the control and sham group (*n* = 6 per treatment)**.

**Variables**	**Control Mean ± *SD***	**Albumen-removed Mean ± *SD***	**Sham Mean ± *SD***
Body mass (wet) (g)	50.19 ± 5.69	47.27 ± 2.21	53.40 ± 2.98
Body mass (dry) (g)	11.59 ± 1.56	10.99 ± 0.67	12.71 ± 1.49
RMR (mLO_2_h^−1^)	124.6 ± 10.01	124.6 ± 27.41	123.16 ± 21.70
RMR (mLO_2_h^−1^g^−1^) wet mass	2.03 ± 0.12	1.93 ± 0.15	2.05 ± 0.35
RMR (mLO_2_h^−1^g^−1^) dry mass	7.09 ± 0.58	6.94 ± 0.60	6.95 ± 0.68
MR 10°C (mLO_2_h^−1^)	272.46 ± 17.54	228.36 ± 30.41	261.38 ± 24.45
MR 10°C (mLO_2_h^−1^g^−1^) wet mass	5.48 ± 0.65	4.84 ± 0.70	4.90 ± 0.48
MR 10°C (mLO_2_h^−1^g^−1^) dry mass	24.00 ± 3.14	20.89 ± 3.59	20.72 ± 2.27
Carcass (g)	41.34 ± 0.45	38,976 ± 0.72	44.12 ± 0.7
Gizzard (g)	3.33 ± 0.58	3.17 ± 0.31	3.67 ± 0.62
Heart (g)	0.60 ± 0.13	0.524 ± 0.08	0.64 ± 0.05
Liver (g)	2.34 ± 0.73	2.19 ± 0.32	2.35 ± 0.20
Intestine mass (g)	2.58 ± 0.66	2.41 ± 0.26	2.62 ± 0.18
Intestine length (cm)	50.06 ± 1.77	49.73 ± 5.15	53.00 ± 5.75

*We not found statistical differences between groups (p > 0.05)*.

## Discussion

To date, the literature regarding the energetic effects of protein deprivation in birds is scarce, and in some respects conflicting. In this study we explored the hypothesis that variations in the content of albumen could generate differences in metabolic features along developmental pre- and post-hatch periods in a precocial bird. Our results revealed that 3 mL albumen removal can result in decreases in metabolic rates and catabolic activities in skeletal muscle and liver, suggesting negative consequences for muscle capacity, which is, however, reversible in post-hatch life.

The proposal that they represent the integration of all metabolic activities (Vermorel et al., 2005; Swanson, 2010) means that measures of metabolic rates, along with metabolic enzymes, potentially represent an important addition to the study of energy allocation in organisms. In fact, they are capable of indicating differences in catabolic activity of diverse tissues with exquisite temporal and spatial resolution. In this study, we analyzed experimentally the metabolic effects of the removal of albumen in a precocial avian model in an attempt to understand the energetic consequences of protein undernourishment in early ontogeny.

### Embryonic metabolism

We analyzed aerobic metabolism during the final 76% of embryonic development of *Gallus gallus*, from E4 to E20, finding RMR-values comparable to those reported in other studies (e.g., in Murray, 1926: 1.83 cal h^−1^g^−1^ and the current study 1.85 cal h^−1^g^−1^ in control near-term embryos). The removal of albumen resulted in a reduction in mass-corrected (by wet and dry body mass and CAM) metabolic rate (MR) from the E16 until the E20 stage. Total MR, however, did not exhibit differences between groups in near-term embryos, a result that is consistent with reports by Finkler et al. (1998) (average of total of absolute metabolic rate = 406 mL O_2_/day, and the current study 391 mL O_2_/day). However, our results differed from that study in that we observed a 1.5-fold reduction in RMR in the albumen deprived group after correction by body mass. Willems et al. (2014b) reported a reduction in the plasma levels of T_4_ in embryos subjected to the extraction of albumen in E20, which suggests a reduction in the metabolic rate, similar to what appears to occur during the final 20% of embryonic life in our study. Based on these observations, our results contrast with former studies (Davis and Ackerman, 1986; Finkler et al., 1998) that regard egg content (i.e., proportions of albumen or yolk) to have no effect on embryo metabolism. Indeed, our results suggest that the difference in mass in the yolk sac at E16 could be due to different metabolic rates, which would imply a differential use of energy resources (i.e., yolk). However, in this study the difference apparently is transient, and occurs only during the period of highest growth rate (i.e., the exponential phase, see Vleck and Vleck, 1980). These results are partially in agreement with Willems et al. (2013), who reported a reduction in the mass of the residual yolk (not absorbed by the embryo), suggesting a compensatory mechanism by which embryos subjected to albumen deprivation consume a greater volume of available yolk, resulting in body mass equal to the control group at hatching. Energy use of precocial birds increases throughout development, paralleling growth (Seebacher et al., 2006), and a reduction in embryonic metabolism could therefore have consequences for wild birds, affecting the energy budget. Toward the end of incubation this budget is used for competing regulatory functions, including maintenance, locomotion, and thermogenesis (Mortola, 2009).

Metabolic differences generated by changes in albumen content were paralleled—and perhaps explained—by variations in oxidative activity of several tissues (Even, 2013). Indeed, some studies have found significant correlations between metabolic rates and metabolic enzyme activities. For instance, Zheng et al. (2013) reported that basal metabolic rates and liver and muscle mitochondrial COX activities were positively correlated in *Pycnonotus sinensis*, and Peña-Villalobos et al. (2014) observed a positive correlation between basal metabolic rates and pectoral CS activity in *Zonotrichia capensis*. In fact, we found that experimentally treated embryos in E20 presented lower enzymatic activities in the skeletal muscles, revealing that oxidative capacity is not a fixed trait throughout development. Accordingly, in the pectoral muscle, we found that total COX was 1.5 times greater in the control group, and enzymatic activity of CS was greater in leg muscles of the control group, through the total activity (1.8-fold). These observations indicate a reduction in the oxidative activity of the muscles, which implies diminished muscle function (Marsh and Wickler, 1982), possibly generated near to E16. A reduction in the muscle function could have many effects, taking into consideration that in birds these tissues are responsible for locomotion and thermoregulation. In line with our results, other studies relating to birds suggest that muscles are an important source of heat in terms of shivering thermogenesis in seasonally acclimatized and cold acclimated birds (Liu et al., 2008; Zheng et al., 2008; Liknes and Swanson, 2011a,b; Swanson and Merkord, 2013; Peña-Villalobos et al., 2014). Therefore, a reduction of this enzymatic activity could have negative consequences in the early development of thermogenic capacity, and therefore survival in wild birds. Similarly, Beauchamp and Harper (2016) suggest that, in mammals, *in utero* undernutrition generates metabolic alterations that may contribute to disease risk.

Few studies have assessed the effect of environmental factors on the activity of metabolic enzymes during embryonic development in birds. Walter and Seebacher (2007) found that reductions in incubation temperatures (e.g., eggs incubated to 35°C, 3° under optimal conditions) might drive increased CS expression in post-hatching chicks, possibly suggesting a PAR hypothesis, oriented to improve the thermogenic capacities in a “colder” world. In our study, the changes in the oxidative capacity of the skeletal muscles (i.e., pectoral and legs muscles) observed in the albumen-removed group apparently do not imply an advantageous or adaptive response. However, we did find an increase in the metabolic activity of liver, both in CS and COX; this result is contrary to the reduction of CS and COX in muscles, and the reduction of RMR. Therefore, our data suggest a trade-off between the oxidative capacities of the tissues in an undernutrition protein context (i.e., reduction in proteins, and albumen). This reallocation of capacity in terms of energy production may be oriented to maintain as priority diverse liver functions at the expense of skeletal muscle capacities (e.g., locomotion and/or thermoregulation). Thus, these differences in enzyme activities, which suggest reallocation of energy and resources, seem to support the IAR hypothesis (Bateson et al., 2004). Furthermore, the higher metabolic activity in embryonic livers of the protein deprived group in this study fits with the results of Willems et al. (2014b), who found hepatic proteome changes in albumen-removed chicks, with a general upregulation of the TCA pathway from the upregulation of several enzymes involved, such as dihydrolipoyl dehydrogenase, aconitate hydratase, and malate dehydrogenase 1. To the best of our knowledge, the trade-off mechanisms observed between metabolic capacities of different tissues are largely unexplored. However, it is likely that they are the result of the effect of competition for stored circulating growth factors, nutrients (e.g., proteins) and other diffusing signals (Simmons and Emlen, 2006).

### Effects of protein undernutrition in the hatching

Our results are in agreement with previous studies that have determined reductions in the hatching success in albumen-removed chicks (see Willems et al., 2014a). It could be argued that physical disturbance and not albumen removal may have caused the observed effect on hatchability. In this vein, it has been reported that physical disturbance of the albumen occurring in the early stages of development could result in embryo mortality (see Willems et al., 2014a for a review). However, at the time of hatching, most of the albumen had already been consumed, thus successful hatching is more likely a factor of the physiological effect of removal of the albumen, for example changes in enzyme activity of skeletal muscles, than physical disturbance *per-se*. We therefore propose a potential explanation for differential hatching success, considering that the enzymatic activity of CS and COX in skeletal muscle (see Figure 4) are considered indicators of locomotors capacities in many taxa (Kohlsdorf et al., 2004; Dymowska et al., 2012). However, while we cannot discard that glycolysis-driven energy generation may be important for these processes, the fiber types present in legs (mainly responsible for hatching) are those characterized predominantly by oxidative metabolism (Ono et al., 1993), which suggests a causal effect: the reduction of oxidative activity in skeletal muscles, especially in legs, explains the reduction in the capacities of breaking and leaving the egg shell, hence reducing the survival of chicks.

In addition, the reduced locomotor capacities for a successful hatch could be a result of the proteolysis observed in muscles from albumen deprived chicks (Everaert et al., 2013), a potential cause of the differences observed in leg mass found in our study. Despite our results in *G. gallus*, studies that have evaluated the survival of chicks following albumen deprivation in eggs also revealed contrasting results within the altricial-precocial spectrum. For example, in *G. gallus* (precocial) as well as the semi-precocial gull species (*Larus michahellis*), a reduction in hatchability before albumen removal was found (see Hill, 1993; Willems et al., 2013). However, no differences in hatching success following albumen removal has been observed in altricial birds, such as *Hirundo rustica* (Ferrari et al., 2006; Bonisoli-Alquati et al., 2008), keeping the eggs within the natural range of variation for proportional albumen content. Thus, these differences may be explained by the differential degree of enzymatic activity (and therefore muscular activity) necessary for successful hatching within the altricial-precocial spectrum (Choi et al., 1993).

### Post-hatch effects

Metabolic rates in the first week post-hatch were similar to those reported in other studies of broiler ontogeny (e.g., ~1.5 mL O_2_ g^−1^h^−1^, Kuenzel and Kuenzel, 1977). Despite the reported changes in RMR and the metabolic enzyme activities as a function of albumen deprivation in *G. gallus* during early ontogeny, no effect in RMR or in organ masses in individuals subjected to the experimental treatment 7 days after hatching was observed. The fact that post-hatch birds did not express any differences in thermogenesis between treatments suggests that experimental animals can compensate for the effect of malnutrition during development by increasing energy balance in an environment with optimal conditions for maintenance (i.e., food *ad libitum*, within the thermoneutral zone).

Finally, our study demonstrated that, in *Gallus gallus*, a variation in egg albumen content has significant effects on the energetic performance of chicks, in particular on mass-specific metabolic rates and metabolic enzyme activities of muscles and internal organs. Such changes, particularly the decrease of citrate synthase in legs muscles, occur simultaneously with an increase in liver metabolism, supporting the IAR hypothesis. Hence, the reduction in the catabolic capacity of skeletal muscle has probably negatively affected the locomotor capacity during hatching. However, our study revealed that all of these metabolic modifications are reversible in the early post-hatching period, indicating remarkable plasticity.

## Author contributions

PS, VP and IP conceived and designed the study. GP and IP performed the experiments. IP analyzed the data with guidance from PS. IP, PS, and VP wrote the manuscript.

## Funding

This work was funded by a Beca Doctorado Nacional (CONICYT) folio: 21130034 to IP, PS was funded by Fondecyt N° 1160115 and CAPES FB0002- 2014 and VP was funded by Fondecyt N° 1140697.

### Conflict of interest statement

The authors declare that the research was conducted in the absence of any commercial or financial relationships that could be construed as a potential conflict of interest.
